# Supplementary material for: Viral commitment to infection depends on host metabolism
Source: eLife. 2026 Aug 20;14:RP107825. doi: 10.7554/eLife.107825 (PMC13493124; doi:10.7554/eLife.107825)
Supplement: Peer review history

# Editor report: eLife Assessment

**DOI:** 10.7554/eLife.107825.3.sa0

**Authors:** Anne-Florence Bitbol

**Affiliations:** Ecole Polytechnique Federale de Lausanne (EPFL) Switzerland

**Keywords:** Convincing, Valuable

This **valuable** study shows the impact of the metabolic state of bacteria on phage infection. The experimental results, based on various phages infecting *E. coli*, are **convincing** and consistent with a two-step adsorption mathematical model. This study should be of interest to the communities working on cell metabolism and on host-pathogen interactions.

# Referee report: Reviewer #1 (Public review)

**DOI:** 10.7554/eLife.107825.3.sa1

In the wild, bacteria can be found in a wide range of metabolic states, including states in which they are resource limited. Because phages heavily rely on the infected cell's molecular machinery to replicate, it is natural to wonder how phage-bacteria interactions depend on the metabolic state of the cell. In this work, Marantos et al. investigate specifically how the rate of infection of 5 different phages changes between cells grown in energy-rich conditions and cells grown in energy-depleted conditions. Their results clearly show that 4 out of the 5 phages studied display a significant reduction in infection rate in cells that are energetically depleted and provide a potential explanation for this observation by looking into the mechanisms that these phages use to irreversibly infect their host cells.

The work also tries to explain the observation using a mathematical/mechanistic model that describes infection as the sequence of two steps, where a phage first needs to bind to a cell receptor, from which it can potentially unbind, and then irreversibly infects by injecting its genome. The mechanistic interpretation offered by the model highlights an interesting trade-off between adsorbing to a metabolically active host and discriminating between active and inactive hosts that, somehow, a phage has to optimize. It would be interesting, in the future, to investigate how different phages optimize this task.

Comments on revised version.

I am happy with how the authors have addressed all the comments. The manuscript is much clearer and more readable and the previous overstated claims have been removed/clarified.

# Referee report: Reviewer #2 (Public review)

**DOI:** 10.7554/eLife.107825.3.sa2

Summary:

The authors investigate the dependence of phage adsorption rates on host metabolic state, using 5 coliphages that differ in their infection cycles and host receptors. They find that four of the 5 phages showed significantly reduced infection under low metabolic states, with phage that generally have weaker adsorption being more strongly affected by low metabolism. The authors complement their findings with a 2-step infection model where phages can disengage from their hosts after initial adsorption. The paper illustrates the power of standardized experimental protocols for quantitative trait comparisons and highlights the dependence of phage infection success on host physiology.

Strengths:

The paper is well written and clearly structured.

The experiments are well designed and particularly commendable is the diligent use of control scenarios to allow for quantitative comparison between phages. This standardized protocol will be valuable for the entire phage community.

The authors convincingly show the impact of host physiology on phage adsorption success. This dependence has so far mainly been considered for intracellular phage replication and the paper shows that host physiology has to be taken into account at all steps of phage infection.

# Referee report: Reviewer #3 (Public review)

**DOI:** 10.7554/eLife.107825.3.sa3

Marantos et al. showed that for some coliphages, the energetic state of the bacterial host cell has a strong impact on whether phage infection is initiated. The authors drew this conclusion from the observation that there are more free phages remaining in the medium after infection of arsenate-azide-treated cells as compared to after infection of untreated cells. These data were analyzed and reported both as ratios of the treated vs. untreated conditions and using a mass-action kinetic model of phage-cell collision in the infection mixture. The data supported the findings that for four phages infecting *Escherichia coli* bacteria, namely, phages λ, 𝜙80, m13, and T6, the phages are less likely to initiate infection if the host bacteria are energy depleted. However, for phage T5, the authors found that their infection propensity is not impacted.

As I have stated in the first submission of this manuscript, the data presented by the authors clearly supported the principal conclusion of the study. The five phages chosen by the authors represent different viral lifestyles and infection mechanisms, highlighting the potential applicability to other *Escherichia coli* phages. Finally, the authors successfully use a classic mass-action model of phage-cell collision to interpret their data. The simplicity of their experimental assay, combined with the use of this mathematical model, offers other investigators who study phage-bacterial interactions in other contexts a potentially useful toolkit to examine infection in general, and specifically, the dependence of phage infection on the host's metabolic state.

Comments on revised version.

In this revised version, the authors have successfully resolved all of my comments. I appreciate that the main text has been majorly revamped, which greatly helps the readers follow the motivation behind the experiment and analyses, and interpret the data. I agree that the revised terminology choice "commitment to infection", instead of the previous interchangeably used "adsorption"/"entry", is much more logical, considering the experimental data. I also commend the authors for writing the modeling part in a very clear, pedagogical, and instructive manner. Overall, I believe that this manuscript will be valuable to those who are interested in phage-bacterial interactions.

# Author comment: Author response

**DOI:** 10.7554/eLife.107825.3.sa4

**Authors:** Anastasios Marantos, Kim Sneppen, Stanley Brown, Namiko Mitarai

**Affiliations:** University of Copenhagen Copenhagen Denmark; University of Copenhagen Copenhagen Denmark; University of Copenhagen Copenhagen Denmark; University of Copenhagen University of Copenhagen Denmark

The following is the authors’ response to the original reviews.

**Public Reviews:**

**Reviewer #1 (Public review):**

In the wild, bacteria can be found in a wide range of metabolic states, including states in which they are resource-limited. Because phages heavily rely on the infected cell's molecular machinery to replicate, it is natural to wonder how phage-bacteria interactions depend on the metabolic state of the cell. In this work, Marantos et al. investigate specifically how the rate of infection of 5 different phages changes between cells grown in energy-rich conditions and cells grown in energy-depleted conditions. Their results clearly show that 4 out of the 5 phages studied display a significant reduction in infection rate in cells that are energetically depleted and provide a potential explanation for this observation by looking into the mechanisms that these phages use to irreversibly infect their host cells.

The work also tries to explain the observation using a mathematical/mechanistic model that describes infection as the sequence of two steps, where a phage first needs to bind to a cell receptor, from which it can potentially unbind, and then irreversibly infects by injecting its genome. While the model is sensible from a mechanistic perspective, the experimental evidence that supports how each model's rate is affected by the cell metabolic state is weak, as only ratios of these rates can be inferred from the data.

**Reviewer #2 (Public review):**

Summary:

The authors investigate the dependence of phage adsorption rates on host metabolic state, using 5 coliphages that differ in their infection cycles and host receptors. They find that four of the 5 phages showed significantly reduced infection under low metabolic states, with phages that generally have weaker adsorption being more strongly affected by low metabolism. The authors complement their findings with a 2-step infection model where phages can disengage from their hosts after initial adsorption. The paper illustrates the power of standardized experimental protocols for quantitative trait comparisons and highlights the dependence of phage infection success on host physiology.

Strengths:

The paper is well written and clearly structured.

The experiments are well-designed, and particularly commendable is the diligent use of control scenarios to allow for quantitative comparison between phages. This standardized protocol will be valuable for the entire phage community.

The authors convincingly show the impact of host physiology on phage adsorption success. This dependence has so far mainly been considered for intracellular phage replication, and the paper shows that host physiology has to be taken into account at all steps of phage infection.

Weaknesses:

There are some concerns about the experimental setup and which conclusions can be drawn from it:

Before phage infection, bacterial cultures are grown to exponential growth, washed, and then resuspended with glucose or arsenate-azide for 10min. It is however, questionable that 10 minutes is enough to simulate high and low metabolic states realistically. 10 minutes seems to be quite short to go from exponential growth to a low metabolic state, given the transcriptional memory of previous environments. It seems more likely that the population will be quite heterogeneous, with cells in various states of transition towards low metabolic states.

While we agree with the reviewer that during metabolic transitions there may be a period in which the population is heterogeneous, with cells in different stages of transition toward a low metabolic state, the 10-minute treatment used here was chosen based on prior work showing that arsenate–azide rapidly inhibits cellular energy metabolism and is sufficient to eliminate the hyper diffusion of the λ receptor (Winther et al., Biophysical Journal 2009, http://dx.doi.org/10.1016/j.bpj.2009.06.027). We have also corrected the DOI for this reference in the manuscript. Furthermore, the ATP pool of log-phase *E. coli* turns over several times per second (Holms et al., Arch. Mikrobiol. 1972, http://dx.doi.org/10.1007/BF00425016). We therefore assumed the bacteria were energy depleted after 10 minutes. We have clarified this point in the revised manuscript.

Given that arsenate and azide inhibit cellular metabolism, i.e., have antimicrobial effects, cells might not just downregulate metabolism but also activate the stress response, and this causes some of the observed effects on phage adsorption. Therefore, the 'low metabolic state' of the cells in this paper could mean that cells are starved or that they are stressed or both.

The reviewer is correct. We don’t exclude indirect effects. However, as nutrients were removed from the bacteria by washing and energy metabolism was inhibited by the addition of arsenate and azide, we assumed a stress response requiring biosynthesis would be unlikely to occur.

The abundance of receptors could change between the high and low metabolic media conditions and contribute to the observed differences in adsorption, while the authors seem to assume in their model that the initial adsorption rate always remains the same.

We do not think that the observed differences in adsorption are explained by a change in receptor abundance. In a previous study using the same experimental protocol as in the present work, phage λ was compared to the metabolically insensitive mutant λh (Brown et al., PNAS 2022, http://dx.doi.org/10.1073/pnas.2106005119). If the lower adsorption in the low-metabolic condition were caused by a reduced number of receptors, then λh should also have shown a lower adsorption rate under the same condition. Instead, no measurable effect on λh adsorption rate was observed. We therefore conclude that the effect is not explained by changes in receptor number on the timescale of the experiment. We have clarified this point in the revised manuscript.

**Reviewer #3 (Public review):**

Summary:

Marantos et al. showed that for some coliphages, the energetic state of the bacterial host cell has a strong impact on whether phage infection is initiated. The authors drew this conclusion from the observation that there are more free phages remaining in the medium after infection of arsenate-azide-treated cells as compared to after infection of untreated cells. These data were analyzed and reported both as ratios of the treated vs. untreated conditions and using a mass-action kinetic model of phage-cell collision in the infection mixture. The data supported the findings that for four phages infecting *Escherichia coli* bacteria, namely, phages λ, ɸ80, m13, and T6, the phages are less likely to initiate infection if the host bacteria are energy-depleted. However, for phage T5, the authors found that their infection propensity is not impacted.

Strengths:

The data presented by the authors clearly supported the principal conclusion of the study ("Viral commitment to infection depends on host metabolism"). The five phages chosen by the authors represent different viral lifestyles and infection mechanisms, highlighting the potential applicability to other *Escherichia coli* phages. Finally, the authors successfully used a classic mass-action model of phage-cell collision to interpret their data. The simplicity of their experimental assay, combined with the use of this mathematical model, offers other investigators who study phage-bacterial interactions in other contexts a potentially useful toolkit to examine infection in general, and specifically, the dependence of phage infection on the host's metabolic state.

Weaknesses:

(1) The authors isolated and measured the numbers of free phages in the medium after infection of bacteria under different treatments. These measurements were analyzed in two different ways: (1) simply as ratios (corrected/normalized using different controls), and (2) fitted using a simple mathematical model. I have concerns regarding both analyses.

(1.1) For the first method, having different time points at which the sample of each phage is collected critically complicates data interpretation. As one incubates the phage-bacteria mixture for a longer time, more infection occurs, and the number of phages collected from the mixture decreases. Therefore, the different incubation time forfeits the goal of "a systematic and quantitative comparison across different phages [...]", just as the authors self-criticized. Conceivably, the authors could have used the shortest measurement time for all phages (i.e., 10 minutes, as for phage λ). Alternatively, the authors could have applied a systematic criterion such as half (or any other fraction) of the latent period of each phage, which would still "maximize the incubation period while ensuring that manipulations were completed before the first infection cycle concluded". In my view, the seemingly arbitrary measurement time for each phage renders the entire first analysis very challenging to interpret. It also goes against the author's proposition that the protocol was "standardized" or "consistent". It is not clear what the readers are supposed to take away from this first analysis, or rather, which evidence, finding, or conclusion the manuscript would lose if the authors only presented the modeling-based analysis.

(1.2) The second method of analysis sought to remove the dependence of the measurements on time. I completely agree with this goal, and the findings extracted from this analysis significantly contributed to the merits of this manuscript. However, the authors achieved this goal using a single time point for each phage to calculate the infection rate (η). As shown in Figure S3, each of the phage depletion curves is anchored by only one data point (note that the P(t)/P(0) = 1 at t = 0 is assumed, not measured). This goes against the typical way this collision model is used in the literature, where a time series is measured and used to fit the model (e.g., DOI 10.1007/978-1-60327-164-6 18, or more recently, PMID 39700139). This practice in the current manuscript reduced the robustness of the inferred η values. This problem is exacerbated by assumptions used by the authors in formulating this model. For instance, the authors used a constant value for the bacterial concentration, B, because "bacterial growth and lysis were negligible" (lines 135-136). However, considering that the bacteria were cultured at 37oC in a very rich medium (first in YT broth, then in 2% glucose), the measurement times of 20, 30, and 55 minutes are most likely one or a few generations of bacterial growth and division.

Related note: I suggest that one of the panels in Figure S3 should be moved to the main text, since it is critical to the second method of analysis.

We would like to clarify that the manuscript does not present two separate methods, but rather one method presented in two steps: a first step with results that are directly tied to the experimental measurements and show whether the effect is present for each phage, followed by a second, analytical step that makes the results comparable across phages.

The first step presents the ratios because they directly reflect the measurements performed in the experiment and allow the reader to see the effect of the metabolic state for each phage in contrast to its control. We agree that these ratios are time-dependent and therefore not suitable for quantitative comparison between phages. Their purpose is to illustrate the experimental outcome and to show that the effect is present (or absent) on a per-phage basis not to compare magnitudes across phages.

We then follow this with the second step, allowing the reader to follow the logic of the analysis. The analytical step that follows does not represent a second method, but a continuation of the same analysis. Here, we remove the time-dependence specifically in order to make comparison of the effect across phages possible, by connecting our results to standard measures such as the adsorption rate η. Importantly, P(0) is measured for every phage in every experiment. The only modeling assumption used (a standard one in the field) is the exponential form for the decay in free phage number, which naturally yields P(t)/P(0) = 1 at t = 0.

Regarding the reviewer’s concern that bacterial growth may not have been negligible over the relevant time window, we note that recent work on rich-to-minimal growth lags in *E. coli* reports substantial delays before growth resumes after nutrient downshift. One 2023 study (Wu et al., Nature Microbiology 2023, https://doi.org/10.1038/s41564-022-01310-w) considering wild-type *E. coli* shows in Fig. 2c a lag of up to about 2 hours after a shift from MOPS minimal medium with 0.2% glucose plus 18 amino acids to the same medium without amino acids. Another 2023 study (Zhu and Dai, Nature Communications 2023, https://doi.org/10.1038/s41467-023-36254-0) examining both rel+ and rel− strains reports a growth lag of about 49 minutes for rel+ and more than 5 hours for the relA deletion strain. While these conditions are not identical to ours, they support the general point that growth does not immediately resume after such shifts. We therefore think it is unlikely that, following transfer from YT, the cells underwent one or a few full generations during the time window of our adsorption measurements.

On the related note: Following the comments of all reviewers on Figure S3, we have decided to remove it to avoid confusion.

(2) The data were able to distinguish phages that successfully infected bacteria and those that remained free in the medium, and the authors appropriately interpreted the data as such throughout the Results section. However, in the Discussion (starting from the very first sentence, line 172), the authors used terms that include "adsorption" and "entry" more interchangeably (for example, see the three sentences in lines 310-313, for "viral entry efficiency is shaped by [...]", then "adsorption kinetics modeling"). I do not see how the authors' data could distinguish between adsorption (the phage particles attaching to the outside of the cell) and entry (the phage DNA being injected into the cell). Conceivably, any phage particles that irreversibly attach to a cell but do not yet inject their genome into the cell would still be removed from the medium and therefore not quantified. Another example: in lines 189-191, the authors interpreted that "[...] when the bacterium is in a low metabolic state, the phage does not bind irreversibly to the host", but how do the authors eliminate the case of no phage binding (i.e., the reversible step) to begin with?

We agree with the reviewer that our use of the terms adsorption, entry, and infection should have been more careful. Our experiment can only identify the irreversible commitment of phage to a host cell. We have therefore revised the text to refer consistently to phage commitment.

Similarly, in lines 283-293, how do the authors delineate whether energy depletion would increase the k_off term or decrease the k_inj term, because either would result in more free phages in the medium as observed in the data? I believe that the writing of the Discussion, as it stands now, is doing a disservice to the conclusions presented in the Results section.

We thank the reviewer for this important point. We agree that the model would work either by k_off or k_inj being dependent on the host metabolic state, and that our original wording was therefore too restrictive. The data do not distinguish between these possibilities; they only constrain the ratio k_off/k_inj. In the revised text, we therefore formulate the argument in terms of this ratio: if energy depletion leads to reduced commitment, this can arise either because k_off increases, because k_inj decreases, or because both change, as long as k_off/k_inj becomes larger in the inactive case. Put differently, what matters is not which individual rate changes, but that the balance between leaving and committing shifts in a way that disfavors commitment to inactive cells. This also leads to the trade-off now discussed in the revised manuscript: efficient commitment to active hosts requires a small k_off/k_inj, whereas strong discrimination against inactive hosts requires this ratio to become significantly larger in the inactive case. Depending on whether this is achieved through changes in k_off or k_inj, the cost of discrimination appears either as slower commitment or as additional energy dissipation. We agree that the previous wording overstated the mechanistic interpretation, and we have revised the Discussion accordingly to bring it in line with what the Results actually support. Based on the comments from all reviewers, we have also revised the terminology throughout the manuscript: instead of error correction, we now refer to this as a discrimination process, and we replaced k_inj by k_com to reflect that our assay resolves irreversible phage commitment rather than DNA injection specifically.

(3) The authors presented an argument that performing infection of all five phages in the same condition is an advantage, allowing for comparison across different phages. While this goal is a completely valid one, it is difficult to reconcile that with the fact that different phages require different optimal conditions for successful infection. For instance, phage T5 famously requires Ca2+ for successful infection into the host bacterium (and later successful replication); see PMID 13174489. However, all infections were performed in TMG, which lacks Ca2+. Perhaps the absence of T5 dependence on the host metabolism is because the infection condition used by the authors was not optimal for T5 to begin with? Similar arguments could be made for other phages.

Our study alone cannot eliminate that possibility. However, we have cited multiple previous studies, for example references citing Braun et al., showing that T5 remains insensitive to the host metabolic state under different buffer conditions. We therefore believe it is unlikely that the lack of metabolic dependence we observe for T5 is simply due to suboptimal infection conditions.

(4) Whereas the manuscript examined five coliphages, only phage T5 and phage λ were discussed extensively. I believe some discussion points for these two phages need clarification.

We focused our discussion on the phages T5, λ and φ80 because these are the phages for which similar effects have been reported previously in the literature. This allowed us to connect our findings directly to existing work and to discuss mechanistic hypotheses in a meaningful comparative framework. For the remaining phages, to our knowledge no prior studies have examined their behavior under comparable metabolic conditions, and therefore a similarly detailed discussion would have been speculative. Nevertheless, all five phages are treated equally in the presentation of the experimental results and in the quantitative comparison of adsorption rates.

(4.1) Phage T5: The data obtained by the authors show that the infection rate of phage T5 is not impacted by the metabolic state of the host cell. Considering that the authors used the terms "infection", "adsorption", and "entry" interchangeably to refer to the irreversible commitment of a phage to a host cell (see point 2), this discussion regarding phage T5 lacks one critical literature context: DNA entry of phage T5 is known to occur in two phases (first-step transfer and second-step transfer). Critically, the second step can only occur if phage proteins encoded by the phage DNA transferred in the first step are expressed (see PMID 10577483 and the cited papers therein). In that context, metabolic poisoning of the host bacteria should have impeded T5 infection. The authors should comment on this point.

As the reviewer pointed out, our usage of the terms infection, adsorption, and entry should have been more careful. Our experiment can only identify irreversible commitment of phage to a host cell. For T5, we expect that this irreversible commitment already occurs upon first-step transfer of phage DNA. As a result, even if second-step transfer is impeded under metabolic poisoning, our method would not resolve that effect. We have added this clarification to the revised manuscript.

(4.2) Phage λ: The experiment using phage λ in this current study shares many resemblances to that in Brown et al. 2022. That feature alone is not a problem, but at many places in the text, the writing is ambiguous as to whether it is discussing the results in Brown et al. 2022 or in the current manuscript. I am giving three examples below, but this is not exhaustive: (i) Lines 67-69, there is no Brown et al. 2022 reference immediately after "a mutant phage variant (λh) could bypass this dependency [...]" (not just in the previous sentence); (ii) Line 228 should clearly say "Our previous findings suggested that phage λ is capable of [...]", since it concerns Brown et al., 2022, not the current study; and (iii) Lines 245-246, there is no Brown et al., 2022 reference immediately after "we observed that a mutant variant [...] even energy-depleted host" (without a reference, it reads like the authors "observed" that finding in this current manuscript).

The reviewer is right. In those places, the text was ambiguous as to whether it referred to the present study or to Brown et al. (2022). We have now inserted the reference at the relevant points and revised the wording where needed to make this distinction explicit.

Also, regarding phage λ: The discussion between line 230 and line 249 is very interesting, but since it concerns the differences between λ PaPa and Ur-λ, the authors should consider mentioning and discussing a very relevant recent study, PMCID: PMC6312755.

We agree that the study by Guan et al. is very relevant and interesting. However, our point in this part of the Discussion is only to clarify that we used λ PaPa and not the originally isolated λ strain. We have therefore limited the discussion here to that distinction.

(5) Control experiments, or references to prior studies, are needed to support that the As/Az treatment at this concentration and duration (at least 10 minutes) is sufficient to deplete the metabolic state of the cell. For instance, this can be shown by impeded or null cell growth, arrested motility (using a standard swimming assay), or a fluorescent reporter for the energetic state of the cell.

The 10-minute treatment used here was chosen based on prior work showing that arsenate–azide rapidly inhibits cellular energy metabolism and is sufficient to eliminate the hyperdiffusion of the λ receptor (Winther et al., Biophysical Journal 2009, http://dx.doi.org/10.1016/j.bpj.2009.06.027) where the effect was assessed by monitoring the rate of movement of the λ receptor on the bacterial surface. We have clarified this point in the revised manuscript.

**Recommendations for the authors:**

**Reviewer #1 (Recommendations for the authors):**

As mentioned earlier, I found the paper interesting and addressed an important and significant knowledge gap.

My biggest concern is about the interpretation of the experimental data in light of the two-step model. In particular, around line 286, it is stated "k_inj is more sensitive to metabolic state than k_off". Assuming k does not depend on metabolic state, which is a fair assumption, the equation for eta only depends on the ratio between k_inj and k_off and not on the individual parameters separately. Consequently, there is no way of saying which one of the two is more affected by metabolic state, unless the model already assumes that k_off is not influenced by metabolic state. The results could equally be explained by k_inj decreasing in metabolically depleted cells, or k_off increasing in such cells. If this is an assumption of the model, this should be clearly stated and not reported as a consequence of the data, as it is at the moment. Also, how does this mathematical model connect to the fitting function used in Figure 2b?

We thank the reviewer for this important point. We agree that the model would work either by k_off or k_inj being dependent on the host metabolic state, and that our original wording was therefore too restrictive. The data do not distinguish between these possibilities; they only constrain the ratio k_off/k_inj. In the revised text, we therefore formulate the argument in terms of this ratio: discrimination requires that k_off/k_inj be larger for inactive hosts than for active hosts, such that commitment is specifically reduced in the inactive case. Put differently, what matters is not which individual rate changes, but that the balance between leaving and committing shifts in a way that disfavors commitment to inactive cells. This introduces a trade-off: efficient commitment to active hosts requires a small k_off/k_inj, whereas strong discrimination requires this ratio to become significantly larger for inactive hosts. If this is achieved through changes in k_off, discrimination comes at the cost of slower commitment by allowing more time to leave; if it is achieved through changes in k_inj, it can preserve fast commitment to active hosts but requires additional energy dissipation in order to actively modulate commitment. We have therefore revised the text accordingly to frame the argument in terms of this trade-off, rather than attributing the effect specifically to k_inj. Based on the comments from all reviewers, we have also revised the terminology throughout the manuscript: instead of error correction, we now refer to this as a discrimination process, and we replaced k_inj by k_com to reflect that our assay resolves irreversible phage commitment rather than DNA injection specifically.

I have a related experimental criticism. The kinetic model presented assumes an exponential decay of free phage, which is a commonly used assumption in the phage literature. Given that the phage types used in this study lyse relatively slowly, it would be good to actually see adsorption curves, in which free phage is measured at different time points between inoculation and lysis. This data would not only provide useful evidence for the kinetic model, but it should also replace what is now in Figure S3, which consists of fitting one experimental point with one line. As it currently stands, Figure S3 is not useful actually misleading.

We appreciate the reviewer’s point. We agree that adsorption curves, in which free phage is measured at different time points between inoculation and lysis, would provide a stronger basis for evaluating the kinetic model. However, we do not have the resources to perform these additional experiments within the scope of the present study. Following the comments of all reviewers on this point, we have therefore decided to remove Figure S3 to avoid confusion.

Finally, it is not clear to me why the quantity "Ratio" has been chosen to be presented in Figure 1, rather than the ratio of estimated adsorption rates eta'/eta, which is much more intuitive for a phage study and contains the same information. I would recommend switching to this choice, unless there is a clear rationale for why the quantity "Ratio" is more useful/effective. Showing eta'/eta would also increase the readability of Figure 1, as it would move the y-axis to a logarithmic scale and better visualize values around 1.

We used “Ratio” in Figure 1 to illustrate the experimental design, controls, and measured quantities directly, as it more transparently reflects the data collected. In the second part of the analysis, where we compare time-independent adsorption rate estimates, we have presented the corresponding values of η′/η as suggested.

Minor comments:

(1) Introduction

Line 31: "... such as nutrient limitation, fluctuating temperatures, and variable energy availability" - if drawing a distinction between energy availability and nutrient limitation, please make explicit what this distinction is. Energy availability seems like a natural consequence of nutrient availability.

While energy and nutrient availability are often linked in *E. coli*, they represent distinct physiological constraints. Nutrient limitation refers to the lack of essential biosynthetic precursors such as nitrogen, phosphorus, or amino acids. Energy availability, in contrast, reflects the cell’s ability to generate ATP and reducing equivalents through metabolic processes. For example, under anaerobic conditions, *E. coli* may have ample nutrients but limited energy production due to the lower efficiency of fermentation compared to aerobic respiration. Thus, energy limitation can occur independently of nutrient limitation.

(2) Results

(a) Whole Section: Please label equations.

All equations have now been labelled in the revised manuscript.

(b) Lines 105 to 114: As stated in Major Comments, I think the clarity of the paper would be improved by introducing the relative adsorption rate here and dropping the concept of Ratio entirely. However, if the authors wish to use Ratio, I would recommend the following:

Lines 105 to 109 are confusing to read because of the number of connectives: "... ratio of free viruses from permissive AND resistant hosts respectively TO the free viruses in buffer under energy-depleted AND energy competent conditions". This would be clearer if each quantity were given an algebraic symbol, and RP, RR, and Ratio were defined through formal algebra, rather than mixed mathematical and sentence notation.

This section has been rewritten for clarity. We now introduce explicit algebraic symbols and define the quantities formally, which removes the ambiguity present in the sentence-only description while retaining the intended meaning.

The chemical names "arsenate" and "azide" should appear in the body of the text before they appear abbreviated in an equation. Please state at this point that these are both metabolic inhibitors, as it is not immediately clear what role they play or why you are using them.

The text has been updated to introduce arsenate and azide by name before the abbreviations are used, and we now explicitly note that they act as metabolic inhibitors.

On line 114, the authors helpfully provide an interpretation of Ratio = 1. It would be useful to provide at the same time interpretations of Ratio >1 and <1, perhaps 2 and 0.5 specifically?

We have added brief explanations illustrating the interpretation of Ratio values greater than and less than 1, including examples of 2 and 0.5.

I would consider giving this quantity a more interpretable name than Ratio. This quantity represents how much a bacteriophage preferentially adsorbs to metabolically active cells, so perhaps "Selectivity" or "Adsorption Bias"?

We intentionally retained the generic term “Ratio”, as this quantity reflects an intermediate experimental measure used to describe the process rather than a newly defined metric. Its purpose is to bridge the experimental observations and the subsequent quantification of effects on the adsorption rate (η).

(c) Lines 117 to 122: the authors sometimes refer to ratios explicitly, "average ratio of around 1.6" and other times say e.g., "a greater than 3 times increase in viral particles". Using more consistent language (saying "Ratio" every time) would be clearer.

We have standardized the terminology in this section and now refer to all fold-changes consistently using “Ratio” to avoid ambiguity.

(d) Figure 1

Phages λ and T6 look like they have ratios less than 1 for resistant cells? If this is true / if the ratio is statistically significantly below 1, please comment.

The ratios for λ and T6 are not statistically different from 1. The apparent deviation is within the standard error of the mean. To make this clearer, we have added the corresponding p-values to Table S2 in the Supplementary Information.

Ratios near 1 are difficult to distinguish from 1, especially in panels A and D. Using a logarithmic scale on the y-axis would make the plots more readable.

Because the values in these panels are not statistically different from 1, changing to a logarithmic scale would not alter the interpretation. We therefore retained the current axis scaling to reflect that there is no meaningful deviation from 1 in these cases.

The data corresponding to individual experiments have no error bars. Given that the number of free virions was determined by plaque assay, which carries an intrinsic sampling error, this uncertainty should be reflected in the plots.

We thank the reviewer for this important comment. Because plaque assays have compound sources of stochastic variation, assigning a per-measurement error bar would risk implying false precision. For this reason, we present the values from each biological replicate directly, and the uncertainty is represented in the statistical summary across replicates. Specifically, for each phage and condition we show the three independent experimental measurements and report the mean along with the standard error of the mean. This approach allows us to represent biological variability without implying a precision that cannot be accurately quantified at the level of single plaque counts.

Similarly, the average value does show error bars, but it is not stated what these error bars correspond to: standard error in the mean, standard deviation of the sample, or combined uncertainty?

The caption has been updated to state that the error bars represent the standard error of the mean.

The resistant bacteria seemed to have ratios close to 1 in all cases. Is this because very few virions adsorbed under both energy conditions?

Resistance is commonly associated with a lack of a surface receptor for the phage (or generally an entry pathway). We use the resistant bacteria as a control group for the effect of the conditions on adsorption. For resistant bacteria, the Ratio should be 1 since virions do not adsorb under both energy conditions. Any slight variations from 1 should come from sampling errors or small heterogeneity in the population.

(e) Figure 2

Please comment on what the error bars here represent. Error bars in Figure 2 A seem to permit negative (or at least zero) values of relative adsorption rate for phages m13 and T6, possibly implying an overestimate of the error? If it is the case that multiple values used to calculate the mean are far apart, possibly showing the values individually through a superimposed swarm plot would be clearer.

This point is now addressed in the Supplementary Information, where we clarify how the error bars were calculated.

(3) Discussion

(a) Line 189: "high metabolic state" is imprecise. Say "energy-competent" to be consistent with earlier language.

To maintain continuity with earlier terminology, we now include “energy-competent” in parentheses alongside “high metabolic state,” while retaining the original phrasing for readability.

(b) Figure 3, population level

Show adsorbed virions physically attached to bacteria, rather than removing them completely from the image, as currently, the implication is that at a high metabolic state, there are fewer virions total, not fewer virions remaining in solution because more are adsorbed. You could go as far as to add a third "after centrifuging" row, showing the adsorbed phages stuck in the pellet and the unadsorbed phages remaining in solution.

Thank you for this suggestion. Figure 3 has been updated to depict adsorbed virions attached to bacterial cells, clarifying that the decrease represents adsorption rather than loss of total particles. This change improves the accuracy and interpretability of the schematic.

(4) Methods and Materials

(a) Figure 5

The step "estimate cell numbers from OD" appears to follow incubating plates overnight. If the cells you are counting come from the pellet produced by centrifuging 3 steps prior, you could add a fork into the black line connecting the steps, with one branch corresponding to the supernatant and phages, and the other to the pellet and cells?

Thank you for pointing this out. The order in the figure has been corrected: cell numbers are estimated from OD before overnight incubation. This resolves the confusion without the need for branching in the workflow diagram.

(a) Line 332

You allow as much time as possible for adsorption without the possibility of lysis. Did you determine the lysis times / latent periods of these phages through one-step-growth-curves, or use published results, in which case please cite? Having obtained the lysis time by either method, what fraction of the lysis time did you allow for adsorption? Also, please add supplementary tables with lysis times used for the different phages.

We thank the reviewer for this comment. We used published latent-period values as guides and verified compatibility with our own system when selecting incubation times. We have clarified this in the text and added the relevant citations. We did not use a common fixed fraction of the lysis time for all phages; instead, incubation times were chosen to allow sufficient time for adsorption but not for completion of the first lytic cycle. For λ, productive lytic development was blocked in the host background used, as in Brown et al., PNAS 2022, http://dx.doi.org/10.1073/pnas.2106005119. For ϕ80 and T5, we used published latent-period values as guides and verified their compatibility with our own system (De Paepe and Taddei, PLoS Biology 2006, http://dx.doi.org/10.1371/journal.pbio.0040193). M13 is a chronic filamentous phage and therefore does not have a standard lytic latent period; in our host–phage combination, it required more than 1 h before phage release. For T6, we relied primarily on the kinetics observed in our own system, since adsorption was unusually slow for this phage–host pair under our assay conditions. Although literature reports describe shorter T6 latent periods under specific assay conditions (Foster and Johnson, Journal of General Physiology 1951, http://dx.doi.org/10.1085/jgp.34.5.529), this is consistent with published work showing that adsorption and infection kinetics can vary substantially with host background, surface structure, and experimental conditions (Heller and Braun, Journal of Bacteriology 1979, http://dx.doi.org/10.1128/jb.139.1.32-38.1979; Storms et al., Biochemical Engineering Journal 2012, http://dx.doi.org/10.1016/j.bej.2012.02.010).

(5) Supplementary

Figure S1

This data is useful in understanding the main body of the paper, and I think this should form part of a main figure (possibly with the individual experimental data points superimposed over the bars). This could come before or as part of Figure 1?

We thank the reviewer for this suggestion. We have explored including these data directly in the main figure but found that doing so substantially reduced the readability of the figure, as the underlying table is visually dense. For this reason, we chose to summarize the results in Figure 1 and present the detailed data separately in Figure S1 of the Supplementary Material, along with the Ratio analysis, which more effectively conveys the trends without overloading the main figure.

**Reviewer #2 (Recommendations for the authors):**

Minor comments:

(1) L16-18: This sentence could be made more accessible as 'error correction' is not an intuitive term in the phage field.

We have updated the overall theory section including the terminology. Instead of error correction, we now refer to it as a discrimination process.

(2) L96-98: Does this potentially indicate a trade-off where evolution for stronger binding cannot evolve at the same time as responsiveness to metabolic activity?

We agree that this sentence made a stronger evolutionary claim than our data support. Since we only tested four laboratory phages, we cannot conclude that there is an evolutionary trade-off between stronger binding and responsiveness to host metabolic activity. We have therefore removed this sentence to avoid making an unsupported evolutionary interpretation.

(3) L102: What does 'post-cellular' mean?

Postcellular supernatant is simply the liquid that remains after cells have been removed. During centrifugation, the cells pellet at the bottom, and the liquid above (which can contain viruses) is the postcellular supernatant.

(4) L105-107: Worth splitting into two sentences as it is a bit unclear if ratios are built between permissible and resistant hosts or between buffers or both.

Thank you for the suggestion. We have rewritten this section into two sentences to clarify how the ratios are constructed, and we hope the revised wording improves readability.

(5) L110-122: Figures S1 and S2 could be referenced here.

References to Figures S1 and S2 have now been added in this section.

(6) L137: As P(0) is the viral concentration in buffer, I am assuming that the phage lysate has been diluted in buffer and phages have been added to cultures from the same dilution tube to guarantee equal starting numbers, but I couldn't find this in the methods.

This clarification has been added to the Methods and Media section of the Supplementary Information.

(7) L243: It would be worth defining what 'hyperdiffusion' means.

We have added a brief definition of “hyperdiffusion”.

(8) L253-256: I do not entirely follow this explanation.

We thank the referee for pointing out this lack of clarity. This was also raised by Reviewer #3. The point we intended to convey is that λ behaves differently toward *E. coli* LamB depending on whether it is on a living cell or isolated in buffer, but makes no such distinction for *Shigella* LamB, binding it in both contexts. More specifically, previous work showed that wild-type *E. coli* extracts could only inactivate λ in the presence of added solvents, whereas control extracts prepared similarly from *Shigella* did not require added solvent for λ inactivation. This observation is consistent with *E. coli* LamB requiring a specific state to irreversibly bind λ. We therefore meant to suggest that the capacity for metabolic-state sensing is not simply a function of phage identity, but also depends on receptor-specific properties that differ between the two bacterial species.

We have rephrased it as follows: Notably, wild-type λ is inactivated by *E. coli* K-12 extracts only when solvents are added, whereas *Shigella* extracts inactivate λ without this requirement (Randall-Hazelbauer and Schwartz, J. Bacteriol. 1973; Schwartz, J. Mol. Biol. 1975; Schwartz and Le Minor, J. Virol. 1975). This suggests that *E. coli* LamB requires a specific state for irreversible binding, a conditionality absent in *Shigella* LamB, indicating that the capacity for metabolic-state sensing may depend on receptor-specific properties.

(9) L284: Why is k_inj necessarily more sensitive to the metabolic state than k_off? Could membrane changes under stress increase k_off?

We thank the reviewer for this important point. We agree that the model would work either by k_off or k_inj being dependent on the host metabolic state, and that our original wording was therefore too restrictive. The data do not distinguish between these possibilities; they only constrain the ratio k_off/k_inj. In the revised text, we therefore formulate the argument in terms of this ratio: reduced commitment in inactive cells can arise through an increase in k_off, a decrease in k_inj, or both, as long as k_off/k_inj becomes larger in the inactive case. What matters is therefore not which individual rate changes, but that the balance between leaving and committing shifts in a way that disfavors commitment to inactive cells. This also underlies the trade-off now discussed in the manuscript: efficient commitment to active hosts requires a small k_off/k_inj, whereas strong discrimination against inactive hosts requires this ratio to become much larger in the inactive case. We have revised the Discussion accordingly to bring it in line with what the Results actually support. Based on the comments from all reviewers, we have also revised the terminology throughout the manuscript: instead of error correction, we now refer to this as a discrimination process, and we replaced k_inj by k_com to reflect that our assay resolves irreversible phage commitment rather than DNA injection specifically.

(10) Figure 1: There seems to be more variation between replicates in phage Lambda than in other phages. Is this caused by receptor number heterogeneity in the population?

Unfortunately we do not have a way to compare receptor number heterogeneity across the different phage receptors in our experiments. We therefore cannot conclude that the larger variation observed for phage λ is caused by receptor number heterogeneity in the population.

(11) Figure S1: There seems to be a significant difference between phage Lambda viability in the two buffers - do the authors have an idea where this comes from?

There is no difference in λ viability between the two buffers. The apparent difference in the figure is due to sampling variability.

(12) Figure S3: Last sentence of the legend probably shouldn't say 'upper'.

Following the suggestions from all of the reviewers we have removed Figure S3 as it created more confusion than clarity.

**Reviewer #3 (Recommendations for the authors):**

(1) The text reads as incomplete in some places. Can the authors please provide clarifications on the following points?

(1.1) Lines 235-256: How do the authors draw a conclusion that "a phage can detect host metabolic status" from a study that used purified LamB receptors (i.e., no live cells with any metabolism) extracted from two different bacterial species (i.e., not a difference in metabolic states)?

We thank the referee for pointing out this lack of clarity. This was also raised by Reviewer #2. The point we intended to convey is that λ behaves differently toward *E. coli* LamB depending on whether it is on a living cell or isolated in buffer, but makes no such distinction for *Shigella* LamB, binding it in both contexts. More specifically, previous work showed that wild-type *E. coli* extracts could only inactivate λ in the presence of added solvents, whereas control extracts prepared similarly from *Shigella* did not require added solvent for λ inactivation. This observation is consistent with *E. coli* LamB requiring a specific state to irreversibly bind λ. We therefore meant to suggest that the capacity for metabolic-state sensing is not simply a function of phage identity, but also depends on receptor-specific properties that differ between the two bacterial species.

We have rephrased it as follows: Notably, wild-type λ is inactivated by *E. coli* K-12 extracts only when solvents are added, whereas *Shigella* extracts inactivate λ without this requirement (Randall-Hazelbauer and Schwartz, J. Bacteriol. 1973; Schwartz, J. Mol. Biol. 1975; Schwartz and Le Minor, J. Virol. 1975). This suggests that *E. coli* LamB requires a specific state for irreversible binding, a conditionality absent in *Shigella* LamB, indicating that the capacity for metabolic-state sensing may depend on receptor-specific properties.

(1.2) Line 270, in the abstract, and in the caption of Figure 4: The authors described the model using terms such as "an error-correction mechanism" or "standard error correction", but there is little explanation. Can the authors clarify what kind of "error" is discussed here, and how it is "corrected"? In the "standard error correction" model, what determines which method of correction is "standard"? If "error correction" is a standard term in phage-bacterial interaction modeling, please provide references.

We agree with the reviewer that our use of the term error correction was not appropriate in this context. The proper term is discrimination process rather than error correction. We have now corrected this terminology throughout the manuscript and clarified the underlying logic in the relevant sections.

(1.3) Line 301: The authors speculated that phage T5 is "better suited to ecological niches", but I am not sure how that is consistent with their data showing T5 is more rampant, that they infect both energy-competent and energy-depleted cells, not just depleted cells. Why "niches", and why are T5 better suited to environments "where energy-limited cells dominate", not just any environment?

We agree that this point was not stated clearly enough. What we intended to convey is that T5 would be at a net disadvantage in a niche containing a mixture of energy-competent and energy-deficient hosts. We have updated the main text accordingly.

(1.4) Line 303, and related to point 6.3. above: Phage λ can also infect and replicate in "starved bacterial cells" (shown in Kourilsky 1974 and Geng et al. 2024, both of which were cited in this manuscript). How do the authors reconcile these reports with the discussion point in line 303, and their data that only phage T5, but not λ, shows insensitivity to the host metabolic state?

Our data do not imply that phage λ is unable to infect starved bacteria. As shown in Kourilsky (1974) and Geng et al. (2024), λ can indeed infect and replicate in nutrient-limited cells. Our results specifically indicate that λ infection under starvation proceeds with a reduced adsorption rate, while T5 maintains the same adsorption rate even when the host is starved. Thus, our conclusion is that T5 is insensitive to the host metabolic state at the level of adsorption, whereas λ is not. We acknowledge that the wording in line 303 may have unintentionally led to confusion, and we have revised this part of the text to avoid that.

(2) The following comments relate to the text and figures in the manuscript. There are many places in the manuscript that could use fine proofreading and copy-editing for clarity and consistency. For example:

(2.1) If I understand it correctly, the equation in between lines 109 and 110 should be clarified using terms such as "Free viral particles after mixing with bacteria in Arsenate and Azide" and "Free viral particles in bacteria-free buffer with Arsenate and Azide". As it stands, it is not clear which terms correspond to conditions where bacteria are present.

The equation has been updated to explicitly indicate which terms refer to mixtures containing bacteria and which refer to bacteria-free controls, so that the correspondence between conditions is now clear.

(2.2) Equations in between line 276 and 283, and elsewhere: Some concentration terms are enclosed in brackets ("[BP]"), while most are not.

This notation has been clarified. We now use “[PB]” specifically to denote the transient phage–bacterium complex, distinguishing it from the product P⋅B. All other concentration terms are written without brackets for consistency.

(2.3) Figure 4 and in equations: "BP" or "PB"?

The notation has been made consistent throughout; we now use “PB” exclusively to denote the phage–bacterium complex.

(2.4) Line 284 and line 286: The "inj" in "k_inj" is sometimes italicized, sometimes not.

The notation has been standardized so that k_inj is now formatted consistently throughout the manuscript, without italicizing “inj.” Also we have replaced k_inj by k_com to reflect that our assay resolves irreversible phage commitment rather than DNA injection specifically.

(2.5) Figure 5: Was the step "Estimate cell numbers from OD" really performed on the next day after the experiment (i.e., >12 hours after infection and phage plating), not immediately after cell washing?

Thank you for pointing this out. The figure has been updated to reflect the correct order of steps: cell numbers are estimated from OD immediately after washing, followed by overnight incubation of the plates.

(2.6) Figure S1: As it stands now, the x-axis of each panel can be read either as "Permissive, Resistant bacteria, Buffer" (missing "bacteria" for the first pair of bars), or "Permissive (bacteria), Resistant (bacteria), Buffer (bacteria)" (extra "bacteria" for the last pair of bars).

The intended interpretation is the second one (permissive bacteria, resistant bacteria, buffer).

(2.7) Figure S3: The panel letters "A" and "B" are missing in the figure. Also, it is not clear why the legend for the five phages and the legend for the measurement times are not combined.

Following the suggestions from all of the reviewers we have removed Figure S3 as it created more confusion than clarity.

(2.8) Strain table in the Methods and Materials: Please write genotypes with italicization, and consistently indicate mutations and deletions with the minus sign superscript or the Δ prefix. Also, for the S3222 strain: Is it really the entire Mal regulon mutated ("Mal-"), or just lamB-? In Brown et al. 2022, it was only the latter.

Genotypes have been reformatted with consistent notation. For S3222, the correct designation is Mal-, as in the SI of Brown et al. 2022. In this case, Mal- is intended as a phenotypic designation rather than a specific genotype, and we have therefore formatted it accordingly, i.e. neither italicized nor written in lower case.
